# Depleted Housing Elicits Cardiopulmonary Dysfunction After a Single Flaming Eucalyptus Wildfire Smoke Exposure in a Sex-Specific Manner in ApoE Knockout Mice

**DOI:** 10.1007/s12012-024-09897-8

**Published:** 2024-07-24

**Authors:** Michelle Fiamingo, Sydnie Toler, Kaleb Lee, Wendy Oshiro, Todd Krantz, Paul Evansky, David Davies, M. Ian Gilmour, Aimen Farraj, Mehdi S. Hazari

**Affiliations:** 1 Curriculum in Toxicology and Environmental Medicine, University of North Carolina –Chapel Hill, Chapel Hill, NC 27599 USA; 2 Gillings School of Global Public Health and School of Medicine, The University of North Carolina at Chapel Hill, Chapel Hill, NC 27599 USA; 3 Oak Ridge Institute for Science and Education, Oak Ridge, TN 37830 USA; 4 Public Health and Integrated Toxicology Division, Center for Public Health and Environmental Assessment, United States Environmental Protection Agency, 109 T.W. Alexander Dr., Research Triangle Park, NC 27711 USA

**Keywords:** Housing, Wildfires, Resiliency, Cardiovascular, Atherosclerosis

## Abstract

Although it is well established that wildfire smoke exposure can increase cardiovascular morbidity and mortality, the combined effects of non-chemical stressors and wildfire smoke remains understudied. Housing is a non-chemical stressor that is a major determinant of cardiovascular health, however, disparities in neighborhood and social status have exacerbated the cardiovascular health gaps within the United States. Further, pre-existing cardiovascular morbidities, such as atherosclerosis, can worsen the response to wildfire smoke exposures. This represents a potentially hazardous interaction between inadequate housing and stress, cardiovascular morbidities, and worsened responses to wildfire smoke exposures. The purpose of this study was to examine the effects of enriched (EH) versus depleted (DH) housing on pulmonary and cardiovascular responses to a single flaming eucalyptus wildfire smoke (WS) exposure in male and female apolipoprotein E (ApoE) knockout mice, which develop an atherosclerosis-like phenotype. The results of this study show that cardiopulmonary responses to WS exposure occur in a sex-specific manner. EH blunts adverse WS-induced ventilatory responses, specifically an increase in tidal volume (TV), expiratory time (Te), and relaxation time (RT) after a WS exposure, but only in females. EH also blunted an increase in isovolumic relaxation time (IVRT) and the myocardial performance index (MPI) 1-week after exposures, also only in females. Our results suggest that housing alters the cardiovascular response to a single WS exposure, and that DH might cause increased susceptibility to environmental exposures that manifest in altered ventilation patterns and diastolic dysfunction in a sex-specific manner.

**Supplementary Information:**

The online version contains supplementary material available at 10.1007/s12012-024-09897-8.

## Introduction

Living conditions are now widely accepted as important determinants of cardiovascular disease (CVD) incidence and progression [1]. While the impacts of biological, behavioral, and genetic risk factors on the progression of CVD [2] have long been appreciated, it has become increasingly clear that psychosocial factors, including stress [3], depression [4], and anxiety [5] are also associated with the onset and progression of CVD. For example, socioeconomic conditions from childhood are inversely associated with CVD risk in adulthood [6], emphasizing how non-chemical risk factors can have long-term effects on human health. The Multi-Ethnic Study of Atherosclerosis found that low-support and disorderly neighborhood environments are associated with a less healthy diet and decreased access to nutritious food [7, 8], a decrease in physical activity [9, 10], and sleep perturbations [11, 12], all of which increase CVD risk [13]. Thus, housing and neighborhoods indirectly affect cardiovascular health, and likely play a role in disease pathology. However, research that examines the impacts of direct housing interventions on the progression of CVD and the biological mechanisms responsible for such effects remains scarce.

Housing and neighborhood status can also affect physiological resiliency to environmental and chemical exposures. For example, wildfire smoke (WS) has been found to disproportionately impact cardiopulmonary outcomes based on measures of community health, such as income, education, and family and social support [14], while air pollution disproportionately affects lower socioeconomic status (SES) communities [15]. Wildfire smoke exposure has also been shown to induce adverse cardiovascular responses, including triggering a pro-atherosclerotic vascular response to WS in mice [16] and an increased prevalence of atherosclerotic plaques and carotid-intima media thickness [17]. In addition, there is significant epidemiological evidence that air pollution, specifically fine particulate matter (PM_2.5_), is associated with atherosclerotic CVD [18]. Worsening climate conditions have prompted an increase in the prevalence and severity of wildfires [19], and as such may increase the likelihood for spatiotemporal juxtaposition of chemical stressors (i.e., WS) with non-chemical stress from inadequate housing and psychosocial perturbances.

Rodent models are a suitable approach to extrapolate human responses considering living condition-induced psychosocial stress causes similar cardiovascular deficits in both [20] and have been used extensively to study cardiovascular physiology and the cardiopulmonary response to air pollutants [21–23]. For instance, spontaneously hypertensive rats experience increased blood pressure and heart rate when social enrichment was removed from their cages [24], indicating that housing can affect baseline cardiovascular function. Similarly, rat models have shown that environmental enrichment can mitigate, and reverse neurocognitive dysfunction caused by developmental lead exposure [25, 26], suggesting that housing can also alter body resiliency to toxicants. However, few studies have focused on characterizing changes in cardiovascular physiology and function from housing enrichment and the ability of housing to modulate resiliency against an air pollution exposure. Our previous work showed that depleted housing causes increased heart rate, incidence of arrhythmias, lower activity levels and ventilatory depression in healthy mice during a single smoke exposure, responses that are ameliorated by housing enrichment [27]. However, the effect of this housing paradigm on physiological perturbations due to underlying CVD and the corresponding cardiopulmonary response to WS remains unknown. Moreover, it is unknown what role sex plays in the paradigm. Thus, the objective of the present study was to identify the effects of depleted (DH) versus enriched housing (EH) on cardiomechanical function and physiology in male and female atherosclerosis-prone mice and characterize the response to eucalyptus WS. We hypothesized that EH would partially reverse the physiological effects of developing atherosclerosis and the resulting ventilatory response to WS in a sex-specific manner.

## Materials and Methods

### Animals

Eight-week-old male and female ApoE (−/−) mice (Jackson Laboratories – Bar Harbor, ME) were utilized in this study. Mice were housed 5 animals per cage with alpha-dri bedding and a 12-h light/dark cycle in polycarbonate cages. These facilities are maintained at 21 °C and 50% relative humidity in our Association for Assessment and Accreditation of Laboratory Animal Care (AALAC)- approved facilities at the United States Environmental Protection Agency (USEPA). The animals were given access to food and water ad libitum, except during the exposures, and were allowed to acclimate for 7-days prior to the beginning of the study.

### Study Design

**Fig. 1:**
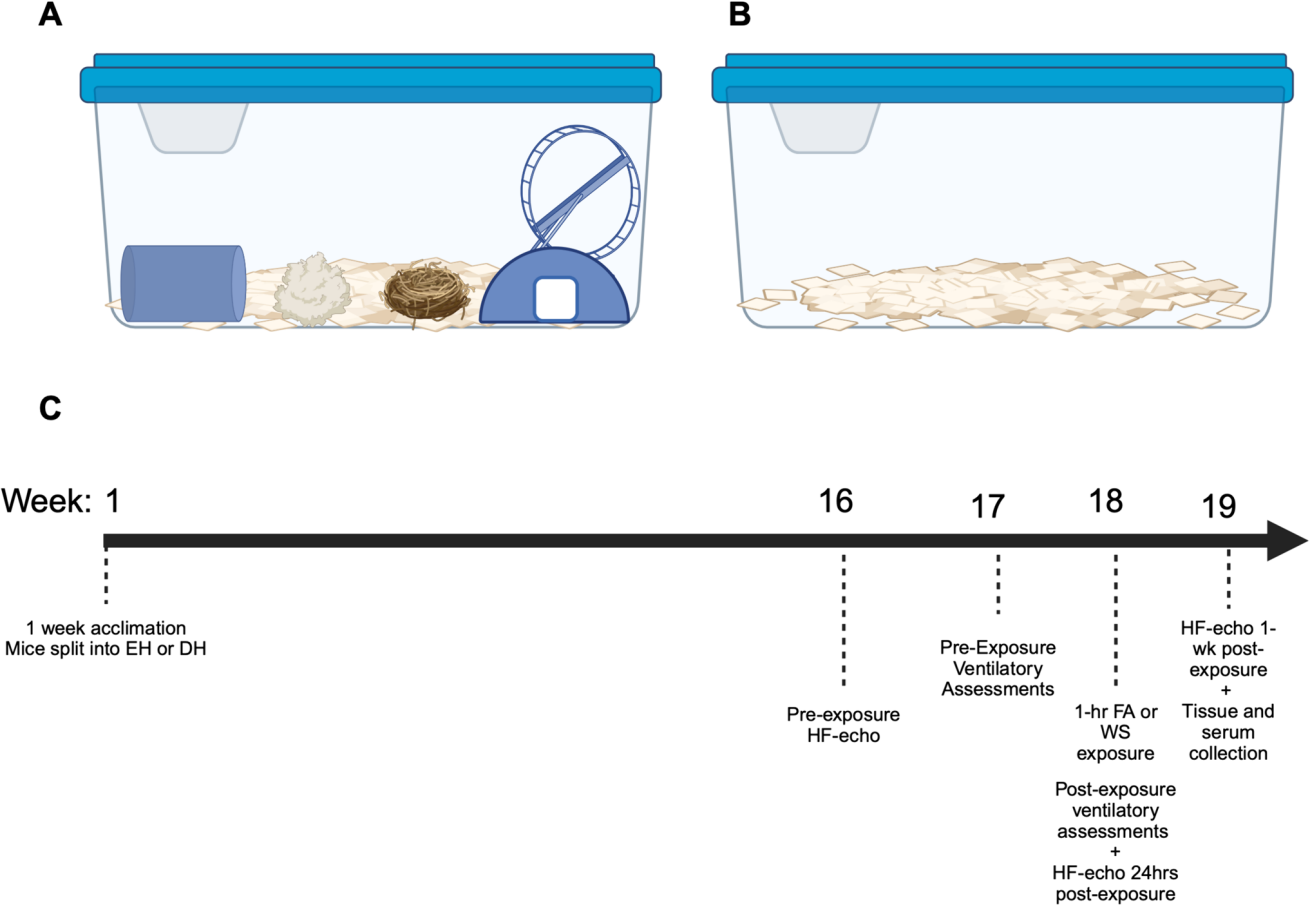
Depiction of housing paradigm and environmental design. **A** Enriched housing (EH) has an alpha-dri bedding with a hut that has an attached running wheel, nestlet, bedding enrichment, and a tunnel, and **B** Depleted Housing (DH) has only alpha-dri bedding in a polycarbonate cage. The mice were split into EH or DH for 18 weeks before being exposed to either a filtered air (FA) sham or a single flaming eucalyptus wildfire smoke (WS) exposure for 1 h (**C**). Pre-exposure and post-physiological assessments were taken to assess ventilatory and cardiovascular function utilizing whole-body plethysmography and high-frequency echocardiography (HF-echo). Figure created with Biorender.com

Mice were acclimated to the facilities for 1 week before being randomly separated into either enriched (EH) or depleted (DH) housing. Enriched housed mice had access to a hut and a wheel, a nestlet (Lab Supply, Durham, NC), a scratchpad, and a tunnel, whereas the DH mice were kept in a bare cage with alpha-dri bedding (Fig. 1). Mice were housed in these conditions for 18 weeks before being randomly exposed once to either 1-h eucalyptus smoke (WS) or a filtered air (FA) sham (*n* = 6/sex/group). High-frequency echocardiography was assessed approximately 1–2-weeks before and 24 h and 1-week after the exposure in order to evaluate the immediate effects from WS-exposure, as well as any long-term interactions between housing and WS, and whole-body plethysmography was performed the week before and immediately after exposure. The necropsy was conducted after 19 weeks, and the mice were approximately 27 weeks old at this time. Serum was collected and the distal aorta was excised and frozen at -80 °C for gene expression analyses.

### Tube Furnace Exposure System for Whole-Body Exposure to Flaming Eucalyptu﻿s Biomass Smoke

An automated control tube furnace system at the USEPA was utilized to create the eucalyptus biomass wildfire smoke under flaming conditions (2 L/min), which has been previously described [28]. Animals were exposed to either the eucalyptus wildfire smoke or a filtered air sham for 1-h in whole-body inhalation chambers (0.3 m^3^ Hinners style stainless steel and glass exposure chamber). Eucalyptus fuel was acquired as writing pen blanks (rectangles at 0.75 inches square by 6 inches long) (Woodworkers source Arizona). A gasoline powered wood shredder (Echo bearcat model number SC3206) was utilized to process the wood and was cleaned in between use. The particulate matter (PM) concentration was monitored continuously and adjusted by a proportional-integral-derivative (PID) feedback loop. Carbon dioxide and carbon monoxide were monitored continuously utilizing a non-dispersive infrared analyzer (Model: 602 CO/CO_2_; CAI Inc., Orange, CA). PM was also collected on a glass fiber filter that was installed in the exhaust line to determine average PM concentrations gravimetrically by weighing the filter before and after the inhalation exposures. The real-time measurements of the wildfire smoke properties and engineering parameters (i.e., temperatures, relative humidity, static pressures, and flow rate) were continuously monitored and analyzed utilizing data acquisition software (Dasylab version 13.0, National Instruments, Austin, TX).

### Whole-Body Plethysmography (WBP)

To assess changes in ventilation patterns, animals were monitored by WBP (Emka Technologies, Falls Church, VA) 1 week before exposure (pre-exposure) and immediately post-exposure, as previously described [29]. The mice were placed in clear plethysmography chambers (3.5″ diameter × 2.5″ height) and given a 5 min acclimation period. Following the acclimation period, data was collected for 15 min and averaged over three five-minute periods, where the following breathing parameters were assessed: inhalation time (Ti), exhalation time (Te), peak inspiratory flow (PIF), peak expiratory flow (PEF), breathing frequency (f), tidal volume (TV), minute volume (MV), relaxation time (RT), and enhanced pause (PenH). Data was collected and analyzed using EMKA iox 2 software (SCIREQ, Montreal, Canada).

### High Frequency Echocardiography (HF-echo)

Cardiac physiology and function was assessed with a high-frequency echocardiography ultrasound system (Vevo 2100, FujiFilm Visual Sonics Inc., Toronto, Canada), as previously described [30]. Animals were anesthetized with 1.5–3% isoflurane delivered in 100% O_2_ at 0.8–1.0 L/min in a sealed whole-body chamber. Once under light anesthesia, the animals were placed on a heated Vevo® Mouse Handling Table (FujiFilm Visual Sonics, Inc.), where isoflurane was continually delivered via nose cone, in dorsal recumbency with each paw grounded to an electrode using Electrode Crème (Cat# 600-0001-01-S, Indus Instruments, Webster, TX, USA) for physiological monitoring and recording of electrocardiogram, heart rate, and respiration rate. An MS-550D transducer was used to image the parasternal long-axis view of the left ventricle using B-mode and M-mode imaging. Pulsed wave Doppler measurements of pulmonary artery and transmitral blood flow was viewed from the short axis and apical four-chamber view, respectively. The sonographer was blinded to the exposure group identities and 3 cine-loops were collected in each view for data analysis.

### Echocardiographic Analysis

Echocardiographic analysis was completed utilizing Vevo® Lab Software (FujiFilm Visual Sonics, Inc.), as previously described [30]. Briefly, while blinded to exposure groups, two beats between breaths for three cine-loops were analyzed. Long-axis M-mode cine-loops were analyzed to measure endpoints related to cardiovascular physiology and cardio-mechanical function, such as, heart rate (HR), cardiac output (CO), stroke volume (SV), fractional shortening (FS), ejection fraction (EF), end systolic volume (ESV), end diastolic volume (EDV), left ventricle anterior wall systole (LVAW;s), left ventricle anterior wall diastole (LVAW;d), left ventricle posterior wall systole (LVPW;s), and left ventricle posterior wall diastole (LVPW;d). Utilizing pulsed wave doppler measurements, we also analyzed blood flow through the pulmonary artery to assess pulmonary artery acceleration time (PAT) and pulmonary artery ejection time (PET). A ratio of these two parameters (PAT/PET) was also calculated. Transmitral blood flow was also assessed utilizing pulsed wave doppler measurements to calculate isovolumic contraction time (IVCT), aortic ejection time (AET), and isovolumetric relaxation time (IVRT). The myocardial performance index was calculated with the following equation: (IVCT + IVRT)/AET.

### Necropsy and Tissue Collection

After the final ultrasound, mice were given an intraperitoneal injection of Euthasol (100 mg/kg Na^+^ pentobarbital 25 mg/kg phenytoin; Virbac Animal Health, Fort Worth, TX, USA). After the animals were unresponsive to a hind paw pinch, blood was collected from the abdominal aorta in serum separator tubes (no anti-coagulant) and centrifuged at 3500 rpm, 4 °C for 10 min. Serum samples were stored at − 80 °C for later analyses with commercially available kits for a KoneLab Arena 30 Clinical Chemical Analyzer (Thermo Chemical Lab Systems, Espoo, Finland). Serum levels of total cholesterol, triglycerides, and glucose were evaluated with kits from TECO Diagnostics (Anaheim, California). High-density lipoprotein (HDL) and low-density lipoprotein (LDL) serum levels were evaluated with a kit from Sekisui Diagnostics LLC (Burlington, MA), and free fatty acids (FFA) serum levels were analyzed with kits from Cell Biolabs, Inc (San Diego, California). The whole heart was weighed, and the distal aorta was excised and frozen in liquid nitrogen and stored at − 80 °C.

### RNA Extraction and Real Time Quantitative Polymerase Chain Reaction (RT- qPCR)

RNA was isolated from ~ 10 mg of aortic tissue from consistent regions of the aortic arch. Direct-zol RNA Miniprep Plus kit (Zymo Research, Irvine, CA) and QIAzol lysis reagent (Qiagen, Valencia, CA) were used to isolate RNA according to instructions provided from the manufacturer. Total RNA quantity and quality (260/280 and 260/230 ratios) was assesses using a Nanodrop 1000 (ThermoFisher Scientific, Waltham, MA). cDNA synthesis using RNA templates was performed using qScript (Quanta Biosciences, Beverly, MA) following manufacturers instructions. Primers were designed using the publicly available NCBI database. Forward and reverse primers were purchased from Integrated DNA Technologies, Inc. (Coralville, IA): *Act-β* f- CTCCCTGGAGAAGAGCTATGA, r- CCAAGAAGGAAGGCTGGAAA; *Vcam-1* f- GAAATGCCACCCTCACCTTA, r- TCTGCTTTGTCTCTCCCAATC; *Icam-1* f- CCAAGAAACGCTGACTTCATTC, r- GGTCTTCTTGCTTGTGTCTACT; *Il-6* f- CTTCCATCCAGTTGCCTTCT, r- CTCCGACTTGTGAAGTGGTATAG; *Ptx3* f- AGGGTGGACTCCTACAGATT, r- TGAGAACCCGATCCCAGATA; *Nampt* f- CCTGACTCTGGAAATCCTCTTG, r- AAGGTGGCAGCAACTTGTA. The relative difference in aortic DNA was quantified through qPCR on a QuantStudio™ 7 system (ThermoFisher Scientific, Waltham, MA) using Sybr Green PCR Master Mix (ThermoFisher Scientific, Waltham, MA) and 15 ng of DNA. Relative gene expression differences were normalized using the ^ΔΔ^CT method and β-actin as the housekeeping gene and DH- FA as the control.

### Bronchoalveolar Lavage

Bronchoalveolar lavage fluid (BALF) samples were collected at necropsy. Room temperature Hank’s Balanced Salt Solution (HBSS) was injected into the lungs via the trachea and repeated for each animal so that there were three aliquots of 0.6 mL of HBSS for analysis. The cells were resuspended in 1.0 mL of HBSS and placed into Coulter vials for total cell counts (Z1 Beckman-Coulter Counter, Miami, Florida). Aliquots of 200 μL were then deposited into Cytospin funnels and spun at 250 rpm for 10 min. The slides were then stained with DiffQuick (RAL Diagnostics) and the number of neutrophils, macrophages, and lymphocytes in the BALF was determined.

### Statistics

All endpoints were analyzed utilizing IBM SPSS Statistics (Version 29.0) using a repeated measures or univariate general linear model in SPSS to assess the main effects and interactions of between subject factors of sex, housing, and exposure and within subject factors of time (for repeated measures analyses), with a Sidak’s adjustment for pairwise comparisons. Pairwise comparisons were only performed when main factors or interaction terms in the overall model were significant. Tukey’s method for outliers was conducted within groups with notable violations of homogeneity of variance, and normality was assessed utilizing a Shapiro–Wilk test. Box cox transformations were performed if needed and in rare cases where normality was still not met, data was analyzed in the same manner as the other endpoints. Findings were considered significant when *p* < 0.05**.** Sex-differences in all parameters are not discussed due to body-mass differences, however, were still performed and can be found in the supplementary material. Graphs were created utilizing GraphPad Prism (GraphPad Software Version 9.0, San Diego, CA).

## Results

### Exposure Characterization

All gas and particle concentrations for the wildfire smoke exposures are in Table 1. The average particulate matter (PM) generated from these exposures was 464.0 ± 340.6 μg/m^3^.

**Table 1:** Average parameters for the flaming eucalyptus wildfire smoke exposures

	PM (µg/m^3^)	CO (ppm)	CO_2_ (ppm)	Smoke temp (°C)	Smoke RH (%)	Smoke flow (LPM)
Eucalyptus WS	464.0 ± 340.6	23.6 ± 16.5	1401.4 ± 414.1	71.1 ± 0.5	53.8 ± 1.1	98.4 ± 0.9

### Body Weight and Bronchoalveolar Lavage (BAL)

While male mice weighed more than females, there were no differences in body weight due to housing across the entire study (Table S1—Supplementary Material). Thus, sex-comparisons for cardiopulmonary parameters (HF-Echo and WBP) are not presented because they likely represent differences in body mass. Other than total cells, the bronchoalveolar lavage also was not significantly different based on housing or exposure (Table S2—Supplementary Material).

### Ventilatory Function

**Fig. 2:**
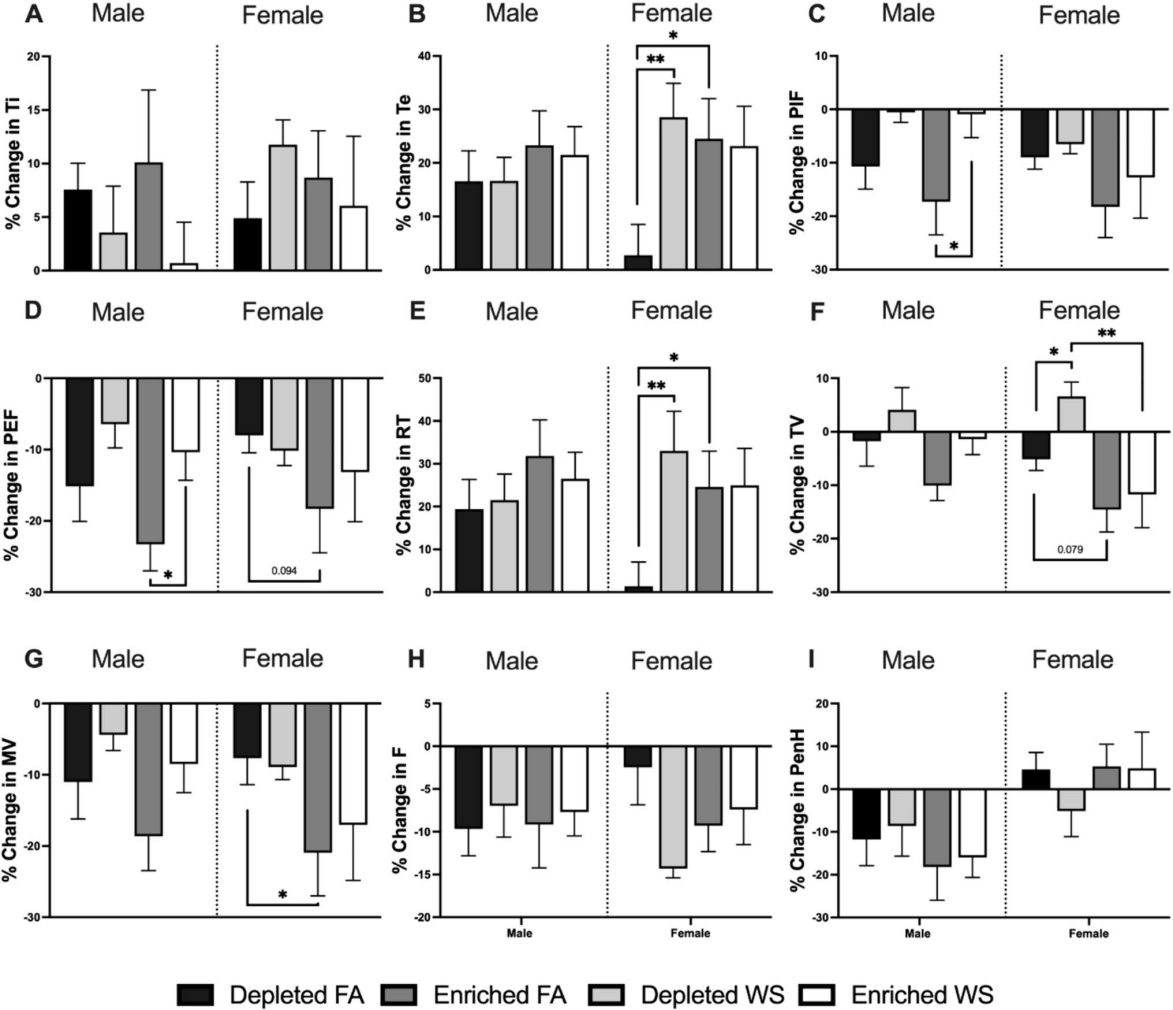
Ventilatory function was measured with whole-body plethysmography (WBP) 1-week before exposures and immediately after the exposures. **A** inhalation time, **B** exhalation time, **C** peak inspiratory flow, **D** peak expiratory flow, **E** relaxation time, **F** tidal volume, **G** minute volume, **H** breathing frequency, and **I** PenH were measured pre-exposure and immediately post-exposure. A percent change calculation from the pre-post-exposure was conducted, and data is reported in a bar graph (*n* = 5–6). * represents significance between groups (*p* < 0.05), ** represents significance between groups (*p* < 0.01), and *** represents significance between groups (*p* < 0.001). *p* values that trend toward significant changes (0.05 < *p* < 0.1) are also indicated

In general, all naïve animals experience a decrease in ventilatory parameters during whole-body plethysmography testing, this is because they eventually relax during the testing period. Regardless, most ventilatory changes (pre-to-post exposure) were observed in female mice, with DH FA experiencing relatively less change with respect to their pre-exposure levels compared to the other FA groups (female and male). WS caused PIF (Fig. 2C) and PEF (Fig. 2D) to be decreased significantly in male EH mice when compared to FA. Although not significant, this also appeared to be the trend with male DH mice. There were no other differences in male mice. Overall, EH prolonged Te and RT (Fig. 2B, E), decreased MV (Fig. 2G), and showed a decreasing trend in PEF and TV (Fig. 2D, F) in all female FA- mice when compared to FA- exposed DH. WS caused Te, RT (Fig. 2E), and TV to significantly increase in female DH mice, this did not occur in female EH mice. While not significant, female DH-FA mice had less decrease in F compared to EH-FA mice (Fig. 2H).

### Cardiovascular Function

**Fig. 3:**
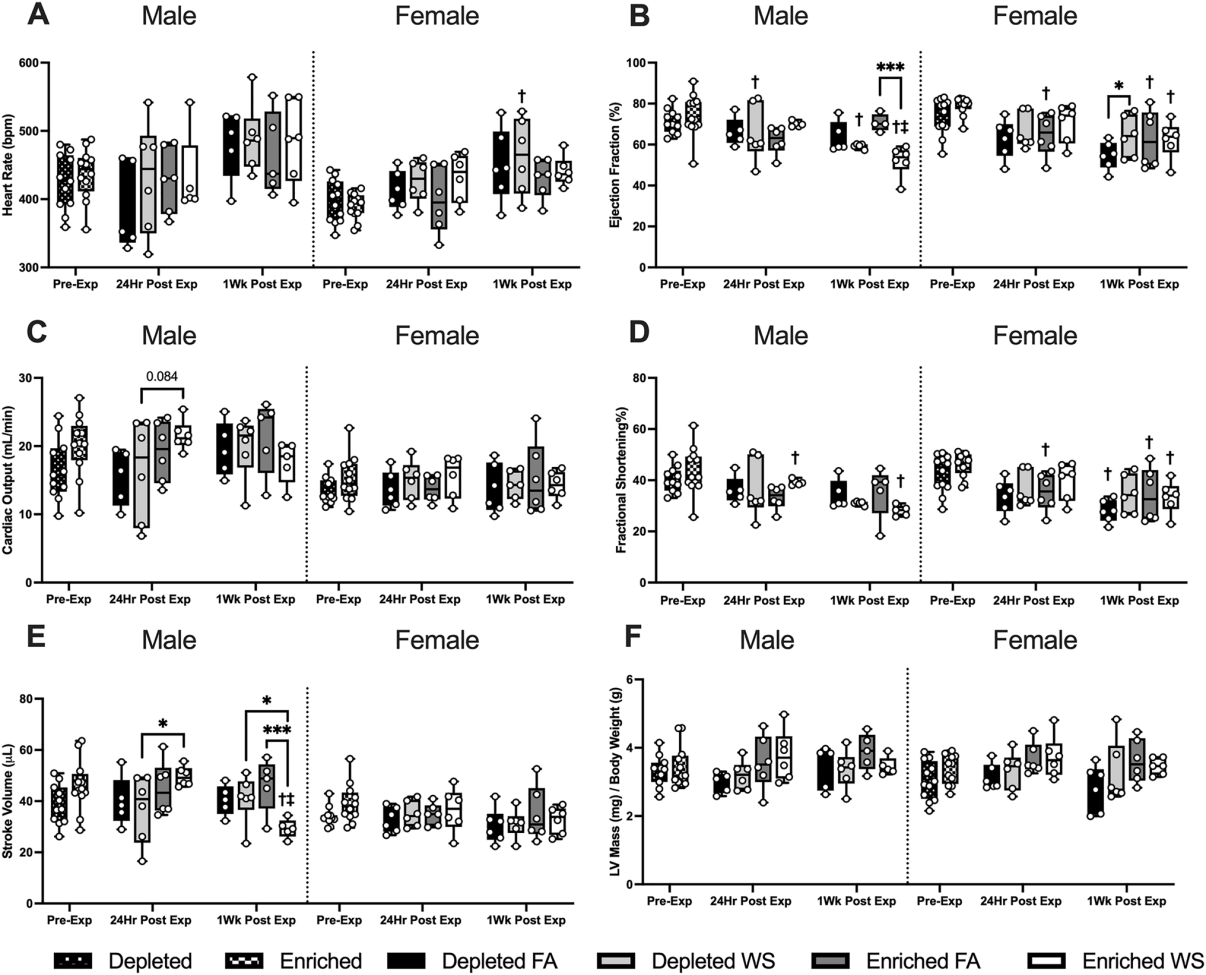
Cardio-mechanical functioning measured utilizing HF-echo. Heart rate (**A**), cardiac output (**B**), stroke volume (**C**), left ventricle mass (mg)/body weight (g) (**D**), end systolic volume (**E**), and diastolic (**F**) were measured pre-exposure, 24 h post-exposure, and 1-week post-exposure. Data is reported using boxplots showing min to max and all points (*n* = 5–6). * represents significance between groups (*p* < 0.05), ** represents significance between groups (*p* < 0.01), and *** represents significance between groups (*p* < 0.001), ^†^ represents significance compared to the pre-exposure timepoint, ^‡^ represents significance compared to the 24 h timepoint. *p* values that trend toward significant changes (0.05 < *p* < 0.1) are also indicated. The magnitude of significance for changes in time are not indicated on the graph and can be found in Supplementary Table 3

**Fig. 4:**
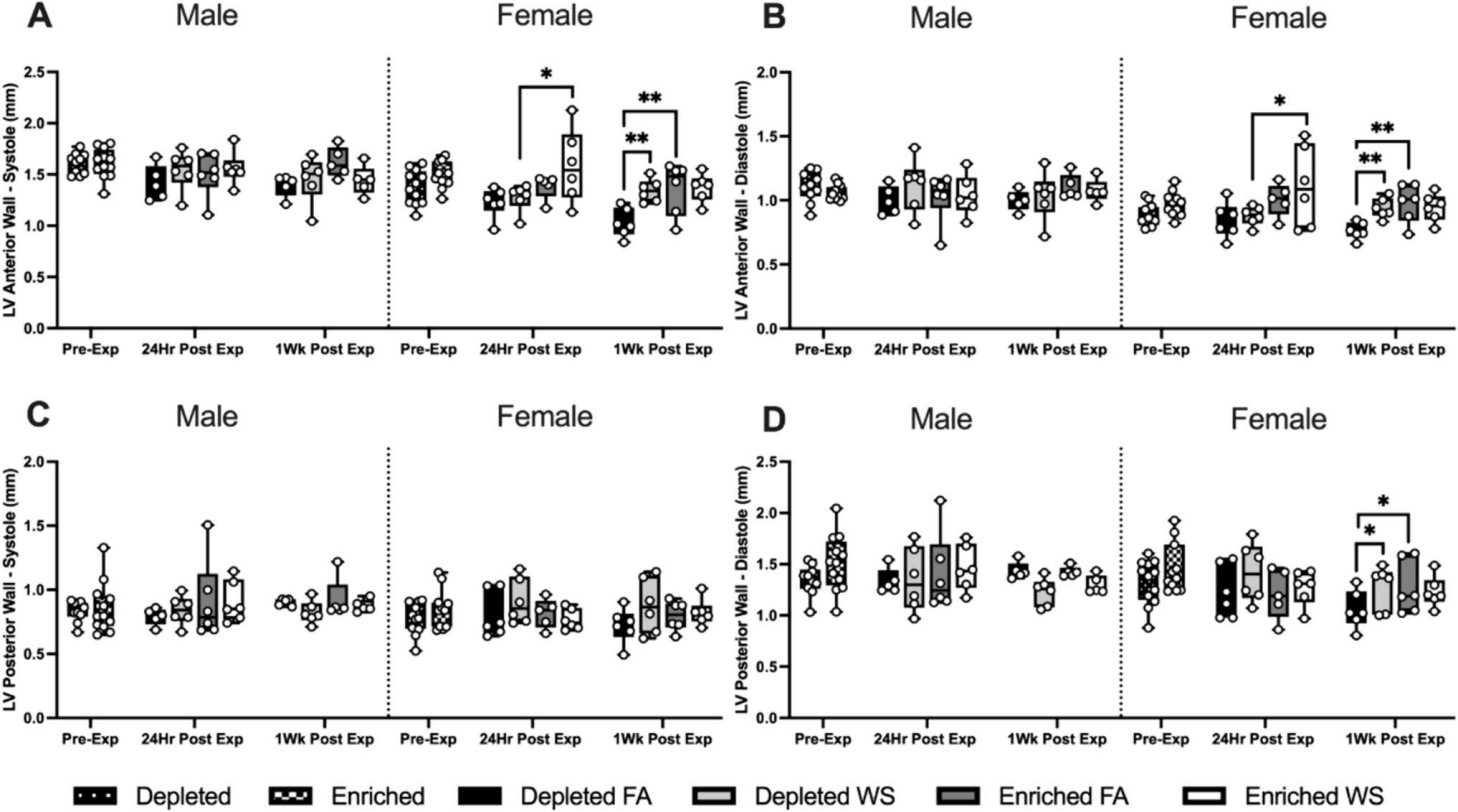
Cardiovascular physiology was assessed utilizing HF-echo. Left ventricular anterior wall size during systole (LVAW;s) (**A**) and diastole (LVAW;d) (**B**) and left ventricular posterior wall size during systole (LVPW;s) (**C**) and diastole (LVPW;d) (**D**) were measured pre-exposure, 24 h post-exposure, and 1-week post-exposure. Data is reported using boxplots showing min to max and all points (*n* = 5–6). * represents significance between groups (*p* < 0.05), ** represents significance between groups (*p* < 0.01), and *** represents significance between groups (*p* < 0.001), ^†^ represents significance compared to the pre-exposure timepoint, ^‡^ represents significance compared to the 24 h timepoint. *P* values that trend toward significant changes (0.05 < *p* < 0.1) are also indicated. The magnitude of significance for changes in time are not indicated on the graph and can be found in Supplementary Table 3

**Fig. 5:**
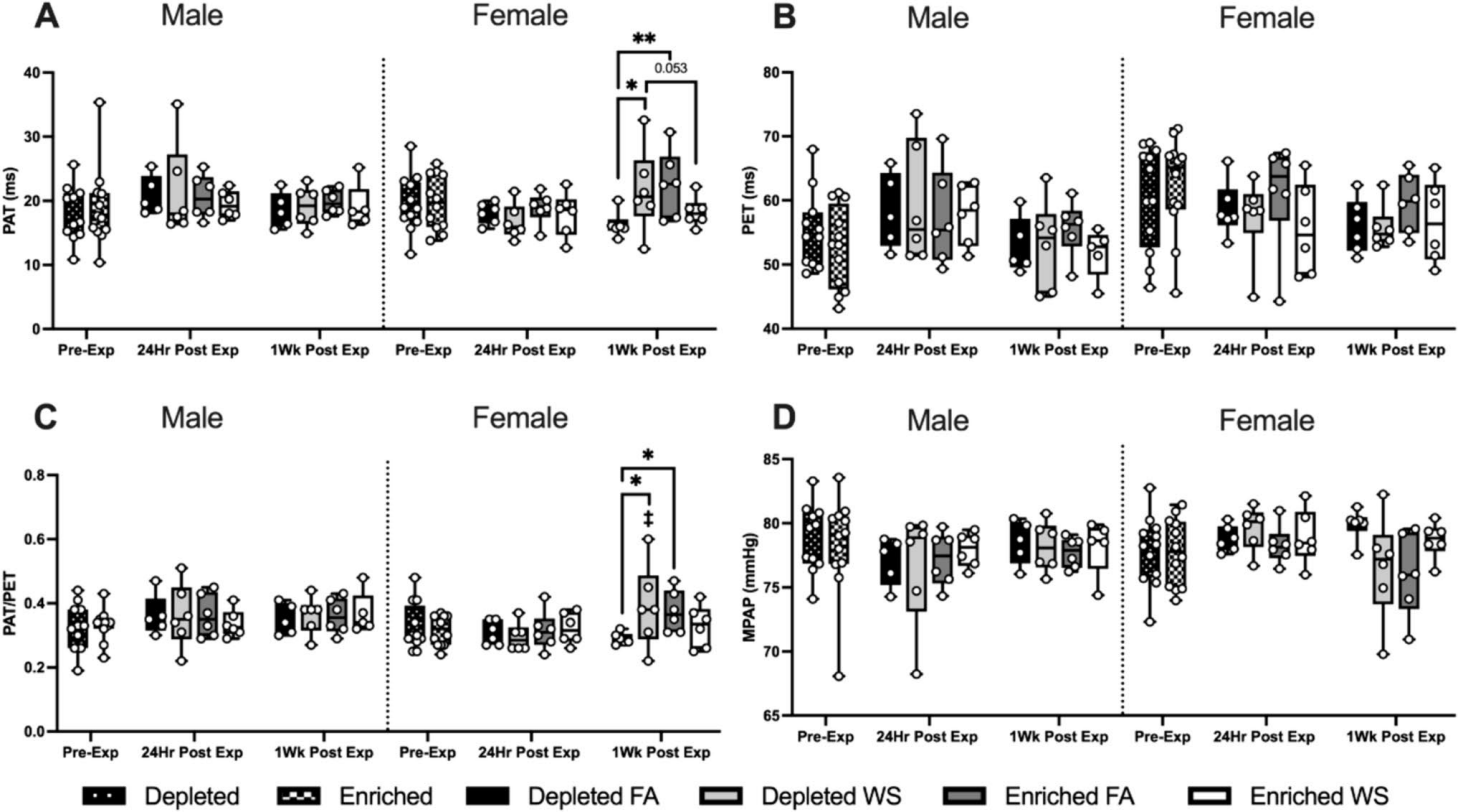
Hemodynamic functioning measured utilizing HF-echo. Pulmonary acceleration time (PAT) (**A**), pulmonary ejection time (PET) (**B**) and the ratio between PAT/PET (**C**) were measured pre-exposure, 24 h post-exposure, and 1-week post-exposure. Data is reported using boxplots showing min to max and all points (*n* = 5–6). * represents significance between groups (*p* < 0.05), ** represents significance between groups (*p* < 0.01), and *** represents significance between groups (*p* < 0.001), ^†^ represents significance compared to the pre-exposure timepoint, ^‡^ represents significance compared to the 24-h timepoint. *p* values that trend toward significant changes (0.05 < *p* < 0.1) are also indicated. The magnitude of significance for changes in time are not indicated on the graph and can be found in Supplementary Table 4

Cardiovascular physiology was assessed 1–2 weeks before (pre-exposure), 24 h and 1-week after exposure. The results are presented to show changes from housing over time, when compared to pre-exposure measurements, as well as between groups to signify effects from both housing and exposure. All statistical analyses, including across sex and all time points, are presented (Tables S3-S5 in the Supplementary Material). There were no differences between DH and EH in both male and female mice at pre-exposure for any parameters (Figs. 3, 4, and 5).

There were no significant differences in HR between any of the groups of male or female mice at 24 h or 1-week post-exposure (Fig. 3A). Male DH-FA mice experienced a decrease (-10.0%) in HR 24 h post-exposure and an increase (24.0%) 1-week later, while male EH-FA had a 0.9% decrease and 8.4% increase at the same time points (Table 2). Male DH-WS mice had a small increase (3.0%) in HR 24 h post-exposure, whereas EH-WS mice had a decrease (-1.1%). HR increased in male DH-WS (13.5%) and EH-WS (8.4%) male mice 1-week later. Female EH-WS mice had an increase of (11.7%) 24 h after exposure, however, 1-week post-exposure this change was not as much (2.1%) (Table 2). Female DH mice experienced a WS-induced increase in HR from pre-exposure to 1-week post-exposure (Supplemental Table 3).

**Table 2:**
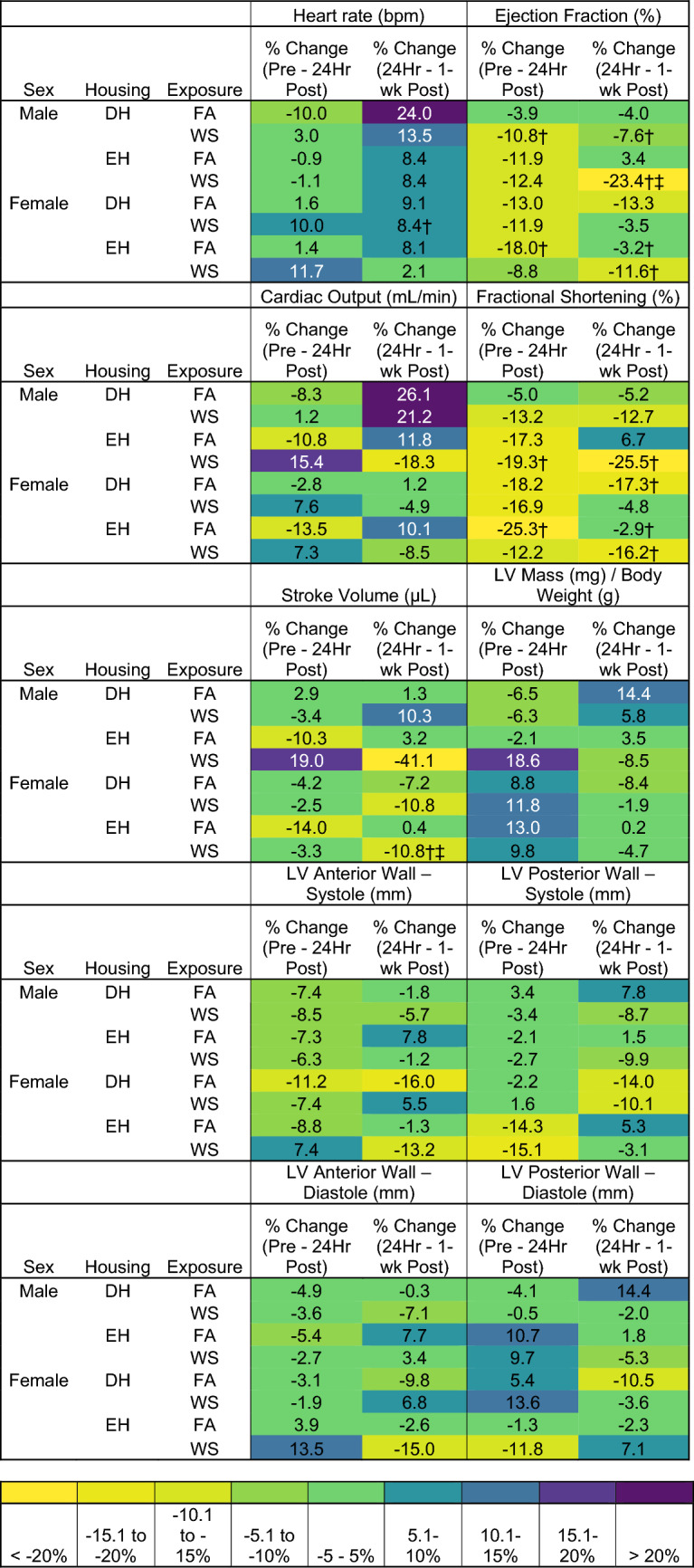
Heat map showing percent changes in HF-echo parameters from Figs. 3 and 4

**Table 3:**
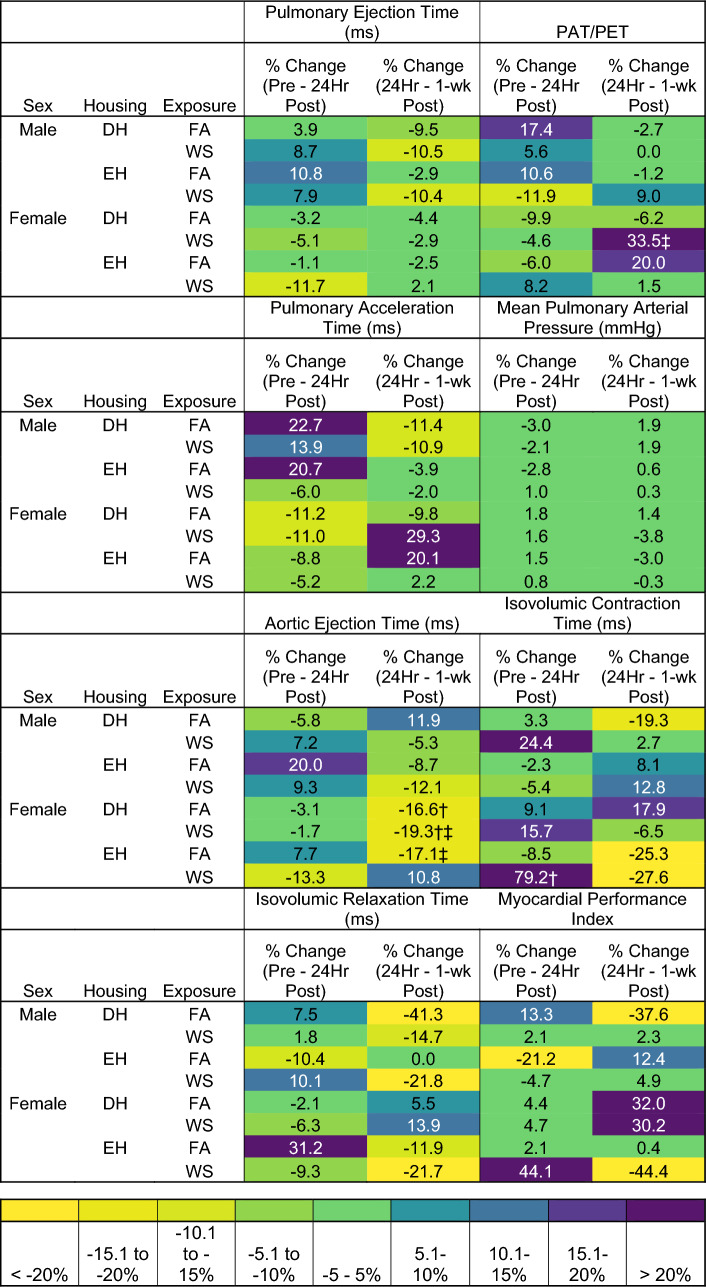
Heat map showing percent changes in HF-echo parameters from Figs. 5 and 6

There was no difference in EF between any of the male or female groups 24 h after exposure (Fig. 3B). However, when compared to pre-exposure, WS caused EF to significantly decrease in male DH mice 24 h post-exposure, and in both male DH and EH mice 1-week after exposure (Table 2), with the response being greater with EH (− 23.0% EH vs. − 7.6% DH). WS-exposed male EH mice had a decrease in EF mice 1-week after exposure when compared to FA (Fig. 3B).

For cardiac output (CO), the only group difference was that WS caused a trend of decrease (*p* = 0.08) in male DH mice 24 h post-exposure when compared to EH (Fig. 3C); there were no other differences in any other male or female groups. It is worth noting, that although the groups did not differ statistically at 24 h or 1-week, there were WS-induced changes in males that differed by housing at both time points. Male EH-FA and DH-FA mice both had decreases in CO (− 10.8% and − 8.3%, respectively), whereas male DH-WS had a slight increase (1.4%) and EH-WS mice had a larger increase (15.4%) 24-h post-exposure. However, 1-week later, male DH mice had an increase in CO regardless of exposure (FA 26.1%, WS 21.2%), and the WS-induced a decrease in male EH mice (− 18.3%) compared to an increase in the EH-FA group (11.8%). In the females, WS-induced changes appear to elicit similar changes in both EH and DH mice, with increases 24-h post-exposure (DH WS 7.6%, EH WS 7.3%), followed by a decrease 1-week later (DH WS − 4.9%, EH WS − 8.5%).

There were no significant differences in FS between any of the groups, male or female, at either 24 h or 1-week post-exposure (Fig. 3D), however there were significant changes in FS over time. Compared to pre-exposure, male EH-WS and female EH-FA mice had significantly decreased FS both 24 h (− 17.3% and − 25.3%, respectively) and 1-week after the exposures (− 25.5% and − 2.9%), while female DH-FA mice also had a decrease in FS only 1-week post-exposure (− 17.3%) (Table 2; Fig. 3). On the other hand, WS caused a significant increase in SV in male EH mice 24 h post-exposure compared to male DH-WS (Fig. 3E). However, 1-week later, the male EH-WS mice had a significant decrease in SV (− 41.1%), which remained significantly lower than both EH-FA and DH-WS mice (Table 2 and S3). Lastly, there were no differences in LV mass between any of the male or female groups, nor was there any significant change over time (Fig. 3F).

There were no differences in EDV and ESV between any groups of male or female mice. However, when compared to pre-exposure, WS caused ESV to increase in male DH mice at 1-week post-exposure and in male EH mice at 24 h and 1-week. In contrast, increased ESV was observed in all female mice exposed to FA at 24 h and 1-week post-exposure (Table S3).

There were no differences in LV anterior or posterior wall thickness between any of the male groups, whether during diastole or systole. On the other hand, WS caused LVAW during diastole and systole to be significantly greater in female EH mice when compared to its effects on DH at 24 h post-exposure. 1-week later, both female EH-FA and DH-WS mice had significantly larger LVAW during diastole and systole and LVPW during diastole than DH-FA (Fig. 4A–D). There were no significant changes across time.

Pulsed-wave doppler imaging assessed blood flow through the pulmonary artery, and imaging of the apical 4-chamber view assessed transmitral blood flow and left ventricular myocardial performance. There were no group differences between the male mouse groups. Hemodynamic changes occurred almost exclusively in female mice, and changes in these parameters are noted at each time point (Fig. 5A–D). WS caused a significantly prolonged PAT and PAT/PET in female DH mice 1-week after exposure when compared to both female DH exposed to FA and female EH mice exposed to WS (Fig. 5A, C). There were no significant changes in PET for groups for both sexes or over time (Fig. 5B). Interestingly, when compared to pre-exposure, all male mice had an increase in PET 24 h post-exposure, followed by a decrease 1-week later, while it decreased at both 24 h and 1-week in the females. WS-induced changes in PAT and PAT/PET in male EH-WS mice, with a decrease in both parameters 24-h post-exposure, and 1-week later an increase in PAT/PET. Similarly, WS-induced an increase in PAT/PET in female EH-WS mice 24 h after exposure. Female DH-WS and EH-FA both had an increase in PAT and PAT/PET 1-week post-exposures (Table 3).

**Fig. 6:**
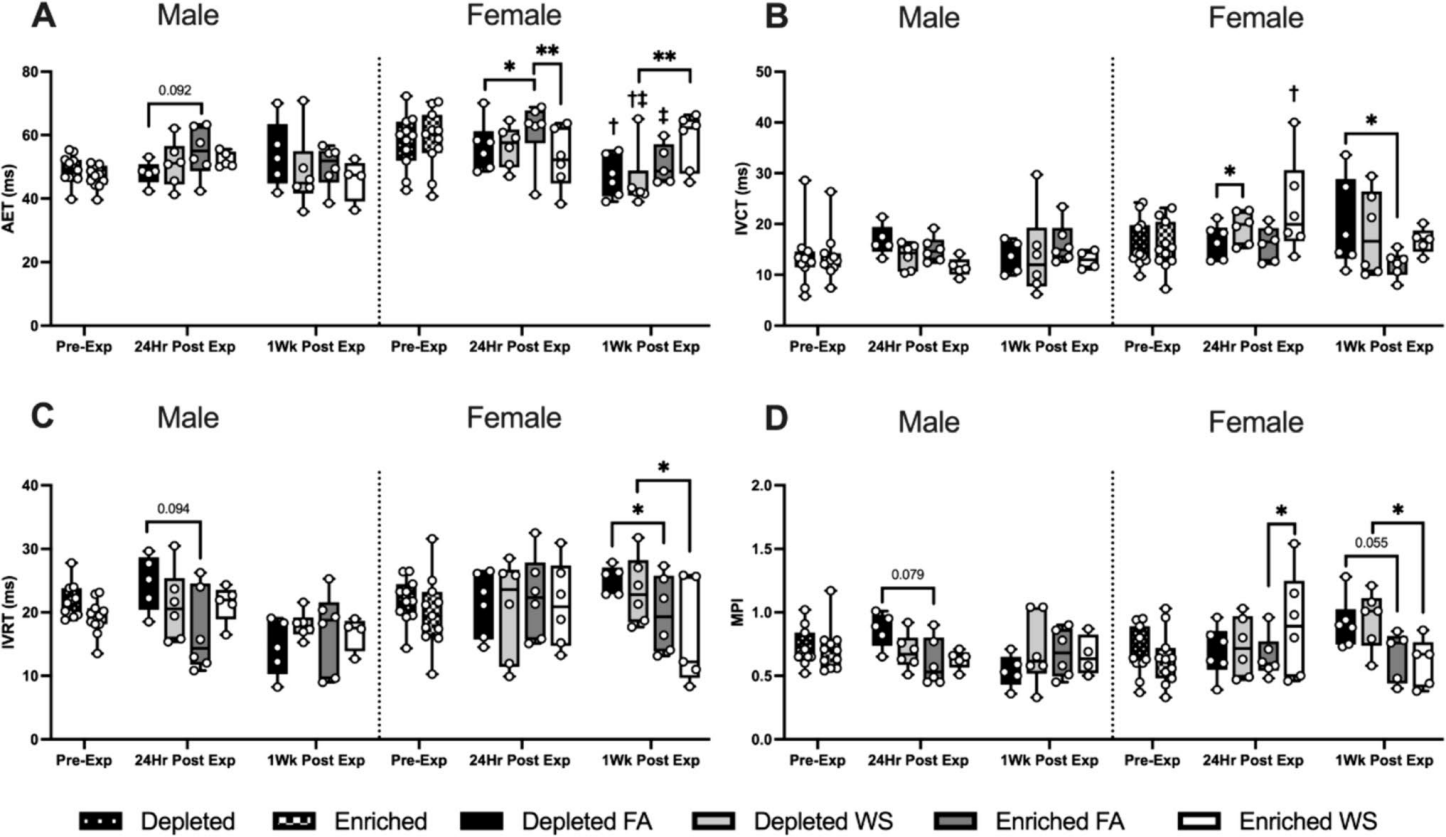
Transmitral blood flow was measured utilizing HF-echo pulsed-wave doppler measurements. Aortic ejection time (AET) (**A**), isovolumic contraction time (IVCT) (**B**), isovolumic relaxation time (IVRT) (**C**), and the myocardial performance index (MPI) (**D**) were measured pre-exposure, 24 h after exposure, and 1-week post-exposure. Data is reported using boxplots showing min to max and all points (*n* = 5–6). * represents significance between groups (*p* < 0.05), ** represents significance between groups (*p* < 0.01), and *** represents significance between groups (*p* < 0.001), ^†^ represents significance compared to the pre-exposure timepoint, ^‡^ represents significance compared to the 24-h timepoint. *p* values that trend toward significant changes (0.05 < *p* < 0.1) are also indicated. The magnitude of significance for changes in time are not indicated on the graph and can be found in Supplementary Table 4

Significant changes between groups in transmitral blood also occurred almost exclusively in female mice, there was a trend toward decreased AET, and increased IVRT and MPI in male DH mice when compared to EH 24 h after FA (Fig. 6A, C, and D). Female EH-FA mice had a significantly increased AET 24 h after exposure when compared to female EH-WS and DH-FA mice (Fig. 6A), and a significantly lower MPI value when compared to female EH-WS (Fig. 6D). On the other hand, WS caused a significantly higher IVCT in female DH mice when compared to FA 24 h after exposure (Fig. 6B). One-week later, WS caused AET to be significantly decreased and increased MPI in female DH mice when compared to EH and had significantly lowered IVCT and IVRT. DH also induced a significant increase in MPI when compared to EH-FA mice, and a trend toward an increased MPI when compared to EH-WS mice.

Furthermore, female DH-FA, DH-WS, and EH-FA all had significant reductions in AET from either pre-exposure or 24 h to 1-week later, while female EH-WS mice had a significant increase in IVCT 24 h after exposures (Table S4).

### Serum Biomarkers

There were few changes in serum biomarkers. Male EH-WS mice had significantly elevated levels of low-density lipoprotein (LDL) and triglycerides, when compared to male EH-FA mice (Fig. S1A, S1D—Supplementary Materials). There were no other significant changes by groups, and there were no significant changes in females for any biomarker.

### Aortic Gene Expression

**Fig. 7:**
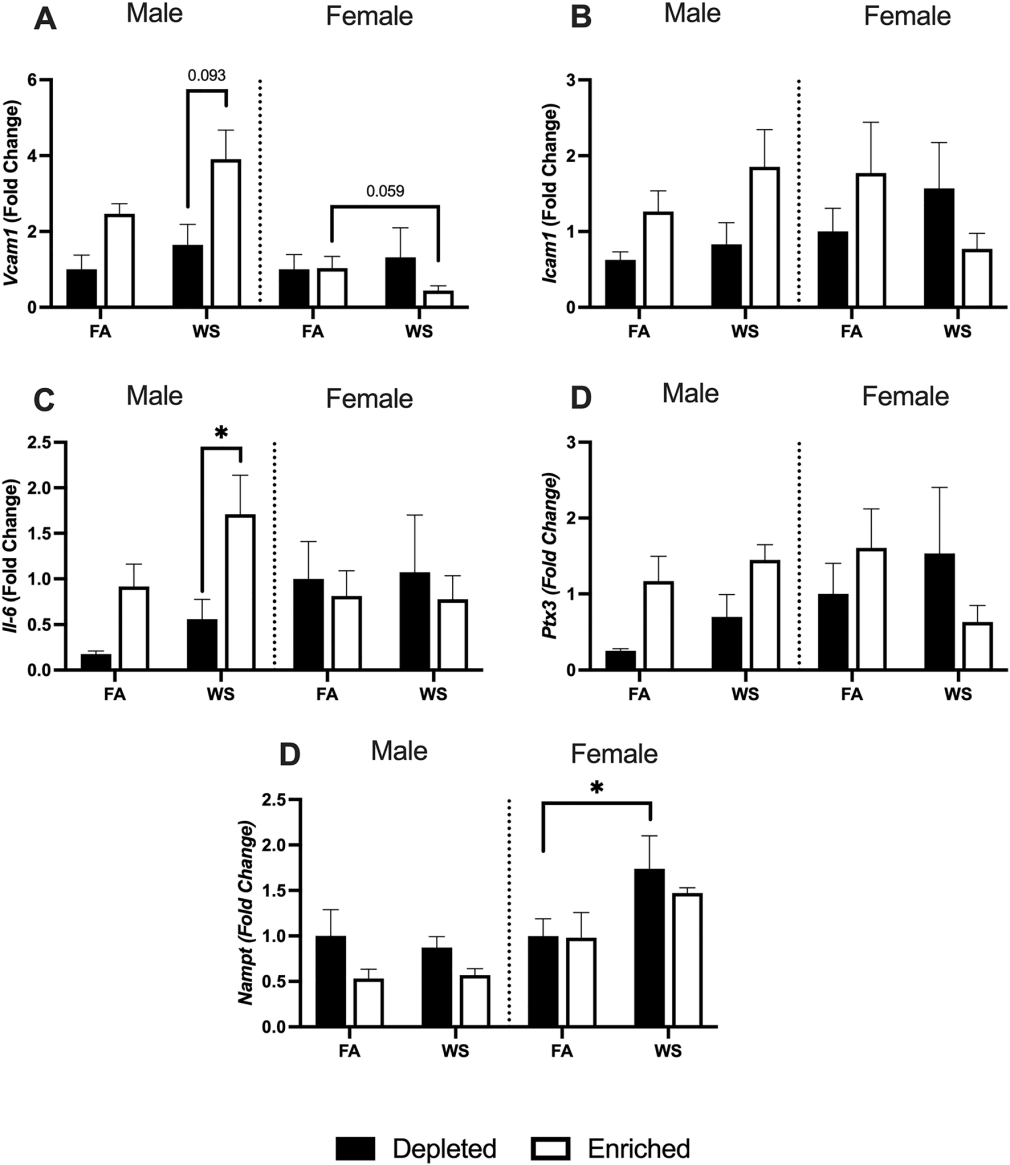
Aortic mRNA expression was assessed utilizing RT-qPCR. *Vcam-1* (**A**)*, Icam-1* (**B**)*, Il-6* (C), *Ptx3* (**D**), and *Nampt* (**E**) were assessed 1-week after the exposures. Data is reported with bar graphs showing all points (*n* = 4–6, ± SEM). * represents significance between groups (*p* < 0.05), ** represents significance between groups (*p* < 0.01), and *** represents significance between groups (*p* < 0.001). *P* values that trend toward significant changes (0.05 < *p* < 0.1) are also indicated

Male EH-WS mice had significantly higher *Il-6* and *Vcam-1* or had an increased trend compared to male DH-WS mice (Fig. 7A, C). Female EH-WS mice had a decreased trend for *Vcam-1* compared to EH-FA mice (Fig. 7A). Female DH-WS mice had significantly increased *Nampt* compared to DH-FA mice (Fig. 7E).

## Discussion

The results of this study show that living conditions, specifically housing enrichment, alter the cardiovascular and ventilatory response to WS, and has potential implications for impacting the progression of disease-related cardiovascular physiology. Psychosocial stressors, such as depleted housing, likely impact the response to wildfire smoke and other chemical stressors by altering cardiopulmonary physiology, and thus, contribute to heightened subsequent adverse effects. While it is clear that wildfire smoke is associated with increased morbidity and mortality [31], the ability of non-chemical stressors to modify the response to these extreme events, especially in the presence of underlying disease remains understudied. We compared enriched housing, which includes several forms of environmental enrichment, with depleted housing to evaluate the role of non-chemical stressors like living conditions in modifying physiological resiliency to a single smoke exposure. Our previous work showed that enriched housing improves cardiovascular outcomes, specifically a lower heart rate, during and after a wildfire smoke exposure and promotes an increase in the expression of cardioprotective genes in the left ventricle [27]. The current study expanded this approach to assess whether housing alters cardiovascular function in atherosclerotic-prone mice and whether the effects are sex-specific. We found that the effect of housing on baseline cardiovascular function and the cardiovascular response to WS in ApoE (−/−) mice occurs in a sex-specific manner. The condition of housing (i.e., depleted vs. enriched) did not cause significant changes in cardiovascular physiology over 16 weeks prior to exposure. However, 24 h and 1-week after a single WS exposure, male mice exhibited changes in cardiomechanical function, specifically, WS-induced a decrease in SV and EF in male EH mice, and a decreased IVRT, and PAT in female EH mice.

The ApoE (−/−) mouse has been a proven model for the spontaneous development of atherosclerosis in both male and female mice [32, 33]. Atherosclerotic lesions occur as early as 10–15 weeks of age, and the lesions grow larger in size and in complexity as the mice continue to age [33, 34]. Moreover, exposure to PM_2.5_ has been associated with an increase in the prevalence of atherosclerotic plagues in male ApoE (−/−) mice [35], increased carotid intima media thickness and prevalence of carotid plaques [17], and an elevated proatherosclerotic response [36]. Coronary artery diseases, such as atherosclerosis, have been shown to adversely impact left ventricular structure, leading to perturbations in left ventricular diastole and myocardial stiffness, seen with increased load and diastolic dysfunction [37].

In human populations, studies have shown that the adverse cardiovascular effects from air pollution can occur in a lag period of up to 40 days [38], which might be mediated by altered neuroendocrine stress axes that can affect immune, inflammatory, and metabolic processes [39], leading to prolonged adverse health effects. Stress has been shown to potentiate this effect by perturbing natural homeostatic function, leading to allostasis and thus an increased susceptibility to chemical exposures [40], such as wildfire smoke. Our results show that while significant changes in cardiovascular function occur in male EH mice 24 h post-exposure, including an increase in SV and a decrease in FS, the physiological response changed 1 week later, with a decrease in SV, FS, and EF. On the other hand, the DH mice exhibit an increase in SV and ESV and a decrease in EF 1-week after the WS exposure. This indicates that the male DH mice might be experiencing an increase in afterload on the heart, or an increase in the pressure needed to eject blood during ventricular contractions, 1-week following the WS exposure. Chronic increases in afterload can lead to concentric hypertrophy and systolic heart failure through a decrease in ventricular compliance and further diastolic dysfunction [41]. Similarly, previous studies have shown that exposure to concentrated ambient PM_2.5_ for 15 weeks elicits reversible cardiac dysfunction through decreases in cardiac output and stroke volume and a concurrent elevation in blood pressure in spontaneously hypertensive rats [42], indicating that housing might provide protection against this type of cardiovascular dysfunction following a WS exposure. While not evaluated in this study, other potential mechanisms that could be causing these cardiovascular changes could include a decrease in cardiac contractility, or inotropy, as well as changes in autonomic function.

Interestingly, female mice did not exhibit changes in the cardiomechanical parameters that were measured (i.e., HR, CO, SV), but rather it was evident in the hemodynamic measurements (i.e., IVRT, MPI). Depleted housing caused an increase in both IVRT and MPI in female mice 1-week after exposures to both FA and WS. An increase in IVRT, or the time from when the aortic valve closes until the mitral valve opens before ventricular filling, may indicate left ventricular diastolic dysfunction that occurs when there is impaired left ventricular relaxation but normal filling patterns [43]. The MPI, also called the Tei index, is a calculated parameter from HF-echo Doppler imaging that evaluates changes in cardiac time intervals, IVCT, IVRT, and AET, to provide information on combined systolic and diastolic function [44]. An increase in the MPI has been shown to be a reliable marker for cardiac dysfunction in congestive heart failure patients [45] and diastolic dysfunction from acute myocardial infarctions [46]. Moreover, WS caused an increase in left ventricular wall measurements 1-week after exposures in female DH mice, but not EH. Increased left ventricular anterior wall measurements, or the intraventricular septum, have been found to be associated with increased systolic blood pressure [47], and increased left ventricular anterior and posterior wall measurements are associated with the incidence of left-ventricular hypertrophy [48]. Although responses vary between sex, WS elicits cardiac dysfunction in both male and female mice, and EH might offer some protection against this dysfunction. Similarly, previous work from our group showed that DH might have potentiated signs of early diastolic dysfunction in male C57BL6/J mice, although there were few cardiomechanical changes seen in females [49]. Thus, this study presents novel information on housing-induced susceptibility to WS in female ApoE (−/−) mice. A possible explanation to these sex-specific changes in pathologies might be due to female ApoE (−/−) mice developing atherosclerotic lesions and plaques faster than the male mice [50], thus the disease progression, and therefore the response to both housing and WS might differ by sex in this model. While atherosclerotic plaque sizes were not measured in this study, future studies should include histopathology of atherosclerotic lesions and other overt measurements of disease progression to further inform this work. In addition, epidemiological studies have shown that women are more susceptible to long-term cardiovascular complications from air pollution compared to men [51, 52], indicating that sex itself might be acting as a biological variable that is leading to the differential changes we see in our study.

Chronic stress can alter lipid metabolism, as well as autonomic and hormonal homeostasis, through activation of the hypothalamus-pituitary adrenal gland (HPA) axis and the sympathetic-adrenal-medullary (SAM) axis [53], which can be a risk factor for atherosclerosis [54]. Our results show that male EH-WS mice have increased levels of triglycerides and serum LDL 1-week after exposures, indicating that there could be alterations in the HPA-axis, leading to lipid dysregulation [55–57]. Similarly, male EH-WS mice also have increased expression of *IL-6* and *Vcam-1* in the aorta, which have been shown to be predictive of peripheral atherosclerosis progression and acute myocardial infarctions [58, 59]. Although our initial hypothesis was that enrichment of living conditions might protect against the development/progression of cardiovascular dysfunction in this model, it is clear that potential worsening of these conditions might not necessarily be ameliorated by psychosocial interventions. Indeed, a more protracted assessment of histological (i.e., aortic plaque) and biochemical changes would clarify this, but the ability of housing enrichment to reverse disease from the perspective of physiological dysfunction was not as evident as expected. We noticed a higher degree of fighting and resource-related aggression in the male EH mice. A previous study has shown that social stress with a male dominant intruder can increase aggression [60]. Thus, there still may have been some amount of psychosocial stress in this group, which contributed to pro-atherosclerotic signs.

Moreover, while both male and female ApoE (−/−) mice spontaneously develop atherosclerosis, the females are generally found to have worsened atherosclerotic development than males even with similar serum lipid and chemistry levels [49]. Interestingly, our results showed that female DH mice exposed to WS might have worsened atherosclerotic progression due to an increased expression of *Nampt*, a gene that is associated with both atherosclerosis and insulin resistance [61, 62]. However, the lack of robust change in serum chemistry markers or gene expression changes in the distal aorta is likely due to the ability of the animals to recover over the 1-week between the WS exposure and tissue collection, and so, it could be argued that measurements in the tissues and serum at an earlier time point might have elicited changes in these biomarkers.

WS is made up of a complex mixture of toxic gases and particulate matter [63] and has been shown to elicit adverse upper and lower airway respiratory conditions [64, 65]. Our results show that housing is able to impact ventilatory outcomes in a sex-specific manner. Breathing frequency was decreased in all groups, as a result of acclimation to the whole-body chamber consistent with our previous findings [66, 67]. WS exposure caused an increase in Te, TV, and RT exclusively in female DH mice, portraying a potential irritant response in the upper airways, indicating that female DH mice might be more susceptible [68, 69]. Women have been shown to be more at risk to adverse responses to ambient air pollution than men [51], and rodent studies have shown that female mice have an elevated response to chronic stress due to increased HPA-axis activity [70], which might be a possible mechanism driving our observations. Further, WS caused an increase in PIF and PEF in male EH mice, but not DH mice. However, there were no significant changes in the inflammatory cells in the bronchoalveolar lavage, likely due to the relatively low WS exposure concentration used and/or the time of the lavage assessments in this study. Other studies from our group have exposed animals to much higher concentrations and have elicited an increased cardiopulmonary and inflammatory response to the WS (PM = 4.2 mg/m^3^ prior study vs. 0.46 mg/m^3^ current study) [28].

In conclusion, housing conditions contribute to sex-specific cardiovascular and ventilatory responses to WS. DH conditions alter the cardiomechanical and hemodynamic response to WS to elicit signs of diastolic dysfunction in male and female ApoE (−/−) mice. Both male and female mice also exhibit alterations in the ventilatory response to WS due to DH, although the response appears to be attenuated in females. Despite this, the cardiovascular changes that were seen from a relatively low PM_2.5_ concentration indicate the ability of housing to alter body resiliency against a chemical stressor. Future work should evaluate the disease progression in both males and females to understand if atherosclerotic-progression could have impacted the sex-specific responses, and if housing can mitigate the advancement of the disease. Moreover, a focus on characterizing the stress response to DH and subsequent activation of the hypothalamic–pituitary–adrenal (HPA) axis and autonomic modulation to evaluate allostatic load, or how housing might modify baseline physiology due to chronic stress, might be beneficial in characterizing the role living conditions play in toxicological risk. Regardless, this work points to the ability of depleted housing as a non-chemical stressor to alter cardiovascular and ventilatory responses to an environmental challenge, with data pointing toward separations in these responses based on sex. Evaluating psychosocial stress as possible modifiers of the toxicological response might be important in determining the health and future-risk to chemical and environmental exposures within human populations.

### Supplementary Information

Below is the link to the electronic supplementary material.

Supplementary file1 (DOCX 653 kb)

## Data Availability

All data for this study is available via USEPA's Science Hub.
