# Supplementary material for: Depleted Housing Elicits Cardiopulmonary Dysfunction After a Single Flaming Eucalyptus Wildfire Smoke Exposure in a Sex-Specific Manner in ApoE Knockout Mice
Source: Cardiovasc Toxicol. 2024 Jul 24;24(9):852–69. doi: 10.1007/s12012-024-09897-8 (PMC11335910; doi:10.1007/s12012-024-09897-8)

Supplementary Material

**Depleted housing elicits cardiopulmonary dysfunction after a single flaming eucalyptus wildfire smoke exposure in a sex-specific manner in ApoE knockout mice**

Michelle Fiamingo^1^, Sydnie Toler^2^, Kaleb Lee^3^, Wendy Oshiro^4^, Todd Krantz^4^, Paul Evansky^4^, David Davies^4^, M. Ian Gilmour^4^, Aimen Farraj^4^, Mehdi S. Hazari^4^

**Table S1.** Body weights expressed as averages ± SD. * represents significance compared to DH. * represents significance between groups (p< 0.05).

| Sex | Housing | Exposure | Week 1 | Week 4 | Week 10 | Week 15 | Week 21 (Necropsy) |
| --- | --- | --- | --- | --- | --- | --- | --- |
|  |  |  | (g) | | | |  |
| Male | DH | FA | 25.62 ± 1.5 | 26.36 ± 1.6 | 31.16 ± 1.9 | 31.54 ± 1.6 | 33.10 ± 1.8 |
|  |  | WS | 25.80 ± 1.4 | 27.15 ± 1.9 | 32.03 ± 1.9 | 31.93 ± 2.7 | 33.45 ± 2.0 |
|  | EH | FA | 27.75 ± 0.4 | 28.93 ± 0.9 | 32.80 ± 1.2 | 32.97 ± 2.1 | 34.87 ± 1.6 |
|  |  | WS | 27.42 ± 0.9 | 28.18 ± 1.2 | 32.10 ± 1.0 | 32.33 ± 2.0 | 33.88 ± 0.9 |
| Female | DH | FA | 20.55 ± 0.9 | 21.55 ± 0.7 | 24.52 ± 0.6 | 24.75 ± 0.6 | 25.9 ± 1.2 |
|  |  | WS | 21.33 ± 1.8 | 21.65 ± 1.1 | 25.58 ± 2.2 | 26.50 ± 2.9 | 27.77 ± 3.6 |
|  | EH | FA | 20.57 ± 1.2 | 21.83 ± 1.4 | 25.95 ± 3.6 | 25.27 ± 1.6 | 25.87 ± 1.6 |
|  |  | WS | 20.58 ± 1.2 | 21.37 ± 1.1 | 24.88 ± 1.4 | 26.22 ± 1.8 | 25.70 ± 1.6 |

**Table S2.** Bronchoalveolar cell differentials expressed as averages ± SD. * represents significance compared to DH, # represents significance compared to the FA control.

| Sex | Housing | Exposure | Total Cells | Macrophages | Neutrophils | Lymphocytes |
| --- | --- | --- | --- | --- | --- | --- |
|  |  |  |  | Cells x 104 |  |  |
| Male | DH | FA | 4.58 ± 1.7 | 4.47 ± 1.6 | 0.0 ± 0.0 | 0.10 ± 0.2 |
|  |  | WS | 4.57 ± 0.8 | 4.44 ± 0.8 | 0.0 ± 0.0 | 0.12 ± 0.1 |
|  | EH | FA | 5.37 ± 1.6 | 5.24 ± 1.7 | 0.0 ± 0.0 | 0.13 ± 0.1 |
|  |  | WS | 6.12 ± 3.0* | 5.94 ± 2.9 | 0.0 ± 0.0 | 0.18 ± 0.2 |
| Female | DH | FA | 4.88 ± 2.8 | 4.76 ± 2.8 | 0.01 ± 0.0 | 0.11 ± 0.1 |
|  |  | WS | 5.91 ± 1.3**^###^** | 5.69 ± 1.3 | 0.05 ± 0.1 | 0.17 ± 0.2 |
|  | EH | FA | 4.31 ± 1.3 | 4.13 ± 1.3 | 0.01 ± 0.0 | 0.16 ± 0.1 |
|  |  | WS | 4.2 ± 1.1***^&&^** | 4.11 ± 1.1 | 0.02 ± 0.0 | 0.10 ± 0.1 |

**Table S3.** HF-echo M-mode data representing cardiomechanical function expressed as a means ± SD (% change from the previous time point). * represents significance compared to DH, # represents significance compared to the FA control, & represents significance compared to the males, † represents significance compared to the pre-exposure timepoint, ‡ represents significance compared to the 24-hr timepoint. * Represents significance between groups (p< 0.05), ** represents significance between groups (p<0.01), and *** represents significance between groups (p< 0.001).

**Table S4.** HF-echo hemodynamic data expressed as a means ± SD (% change from the previous time point). * represents significance compared to DH, # represents significance compared to the FA control, & represents significance compared to the males, † represents significance compared to the pre-exposure timepoint, ‡ represents significance compared to the 24-hr timepoint. * Represents significance between groups (p< 0.05), ** represents significance between groups (p<0.01), and *** represents significance between groups (p< 0.001).

|  |  |  |  |  |  |  |  |  |  |  |
| --- | --- | --- | --- | --- | --- | --- | --- | --- | --- | --- |

Figure S1. Serum Biomarkers expressed as means ± SEM. * Represents significance between groups (p< 0.05), ** represents significance between groups (p<0.01), and *** represents significance between groups (p< 0.001).

|  |  |  |  |  |  |  |  |  |  |  |
| --- | --- | --- | --- | --- | --- | --- | --- | --- | --- | --- |


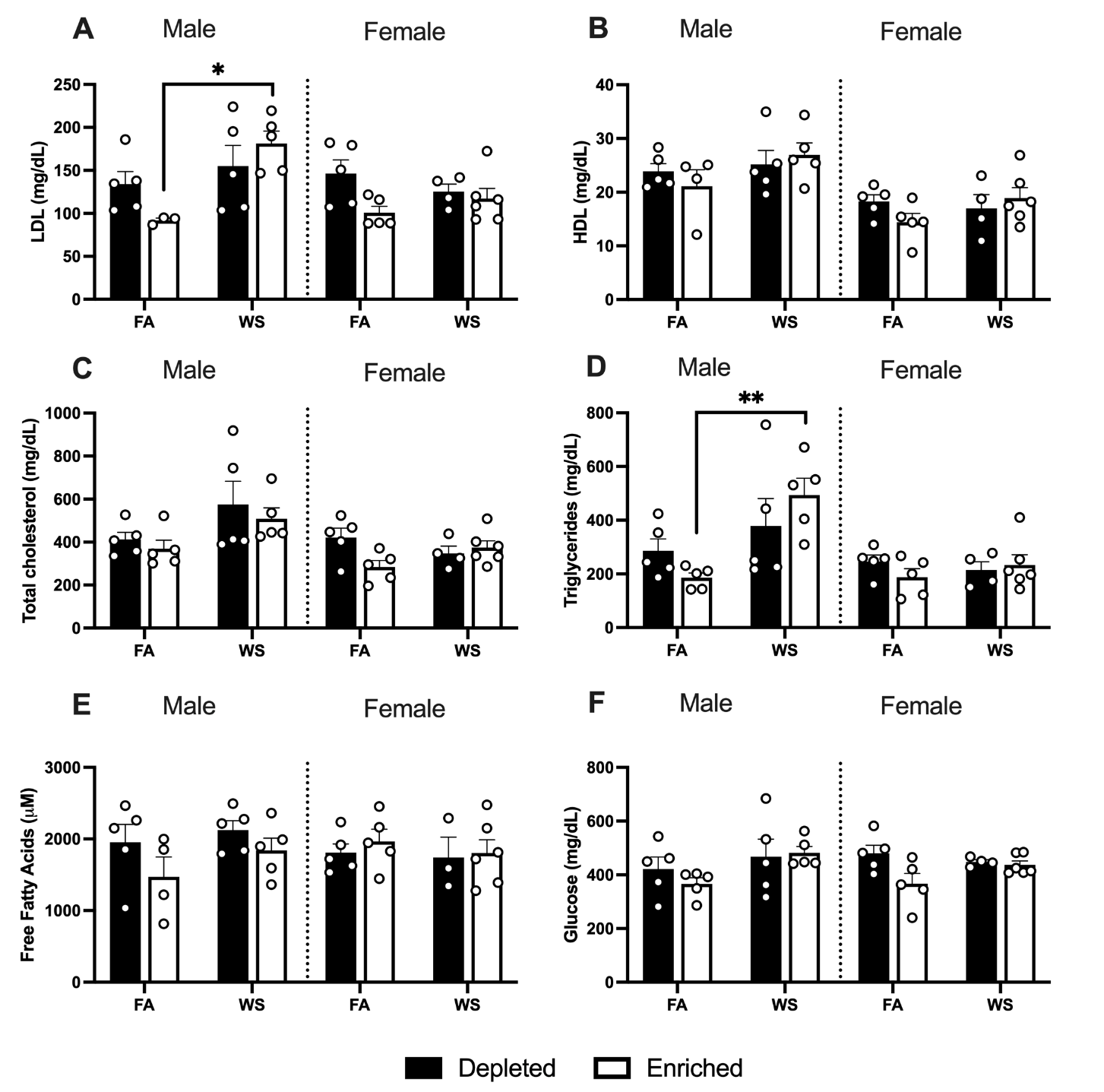

Supplement: Supplementary file 1 — Supplementary file1 (DOCX 653 kb) [file 12012_2024_9897_MOESM1_ESM.docx]
